# Platelet Reactivity and Inflammatory Phenotype Induced by Full-Length Spike SARS-CoV-2 Protein and Its RBD Domain

**DOI:** 10.3390/ijms232315191

**Published:** 2022-12-02

**Authors:** Alan Cano-Mendez, Nallely García-Larragoiti, Maria Damian-Vazquez, Patricia Guzman-Cancino, Sandra Lopez-Castaneda, Alejandra Ochoa-Zarzosa, Martha Eva Viveros-Sandoval

**Affiliations:** 1 División de Estudios de Posgrado, Facultad de Ciencias Médicas y Biológicas “Dr. Ignacio Chávez”, Universidad Michoacana de San Nicolás de Hidalgo, Morelia 58000, Mexico; 2 Programa Institucional de Doctorado en Ciencias Biológicas, Universidad Michoacana de San Nicolás de Hidalgo, Morelia 58000, Mexico; 3 Centro Multidisciplinario de Estudios en Biotecnología, Facultad de Medicina y Veterinaria y Zootecnia, Universidad Michoacana de San Nicolás de Hidalgo, Morelia 58000, Mexico

**Keywords:** platelet activation, platelet aggregation, SARS-CoV-2, spike protein, RBD domain

## Abstract

A state of immunothrombosis has been reported in COVID-19. Platelets actively participate in this process. However, little is known about the ability of SARS-CoV-2 virus proteins to induce platelet activity. Platelet-rich plasma (PRP) was incubated with spike full-length protein and the RBD domain in independent assays. We evaluated platelet activation through the expression of P-selectin and activation of glicoprotein IIbIIIa (GP IIbIIIa), determined by flow cytometry and the ability of the proteins to induce platelet aggregation. We determined concentrations of immunothrombotic biomarkers in PRP supernatant treated with the proteins. We determined that the spike full-length proteins and the RBD domain induced an increase in P-selectin expression and GP IIbIIIa activation (*p* < 0.0001). We observed that the proteins did not induce platelet aggregation, but favored a pro-aggregating state that, in response to minimal doses of collagen, could re-establish the process (*p* < 0.0001). On the other hand, the viral proteins stimulated the release of interleukin 6, interleukin 8, P-selectin and the soluble fraction of CD40 ligand (sCD40L), molecules that favor an inflammatory state *p* < 0.05. These results indicate that the spike full-length protein and its RBD domain can induce platelet activation favoring an inflammatory phenotype that might contribute to the development of an immunothrombotic state.

## 1. Introduction

The Severe Acute Respiratory Syndrome Coronavirus 2 (SARS-CoV-2) is an enveloped positive-sense single-stranded RNA virus that causes Coronavirus Disease 2019 (COVID-19) and has spread worldwide, causing predominantly respiratory illness [1,2]. The SARS-CoV-2 genome encodes a total of 16 nonstructural proteins (nsp 1-16) and four structural proteins: spike (S), envelope (E), membrane (M), and nucleocapsid (N) [3]. The S protein is located on the surface of SARS-CoV-2, with a molecular weight of around 180 kDa, and belongs to a type I transmembrane protein family [4] organized in two subunits: an N-terminal S1, and a C-terminal S2. Viral entry depends on an engagement between the Receptor Binding Domain (RBD), located in the S1 subunit of the protein, and the angiotensin-converting enzyme 2 (ACE2) as the entry receptor [5,6]. The RBD is composed of a sequence of 200 amino acids and is now characterized as a site with a vast number of mutations related to all the novel SARS-CoV-2 variants [7,8]. Critically ill COVID-19 patients show acute respiratory distress syndrome (ARDS), accompanied by a hemostasis imbalance characterized by an active coagulation process and platelet activation associated with systemic inflammation (cytokine storm) in a process defined as immunothrombosis [9]. Platelets are circulating anucleate cells of the bloodstream, traditionally associated with hemostatic processes through their rapid response to vascular damage [10]. Recently, platelets have been positioned as sentinel cells of the immune system due to their unique structural, functional, and generational characteristics, and have been shown to be key mediators in thrombosis and inflammation [11,12,13,14]. In addition, platelets can directly interact with viruses and participate in the immune response, since platelets express a wide range of receptors that they use to interact with pathogens [15,16,17]. Different platelet receptors involved in the recognition of SARS-CoV-2 have been proposed to lead to a state of platelet hyperactivity and an increase in aggregation and adhesion [9,18,19,20,21]. The role of SARS-CoV-2 spike protein and its domain in the platelet–virus interaction, as well as its ability to promote the development of a proinflammatory and prothrombotic state, requires further clarification. The aim of this study was to evaluate the platelet response to SARS-CoV-2 spike protein and domains. 

## 2. Results

### 2.1. SARS-CoV-2 Spike Full-Length Protein and RBD Domain Induce Platelet Activation

**Figure 1:**
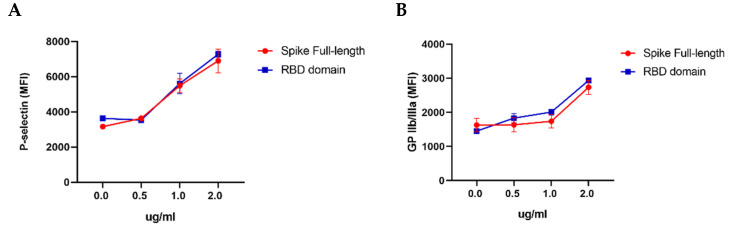

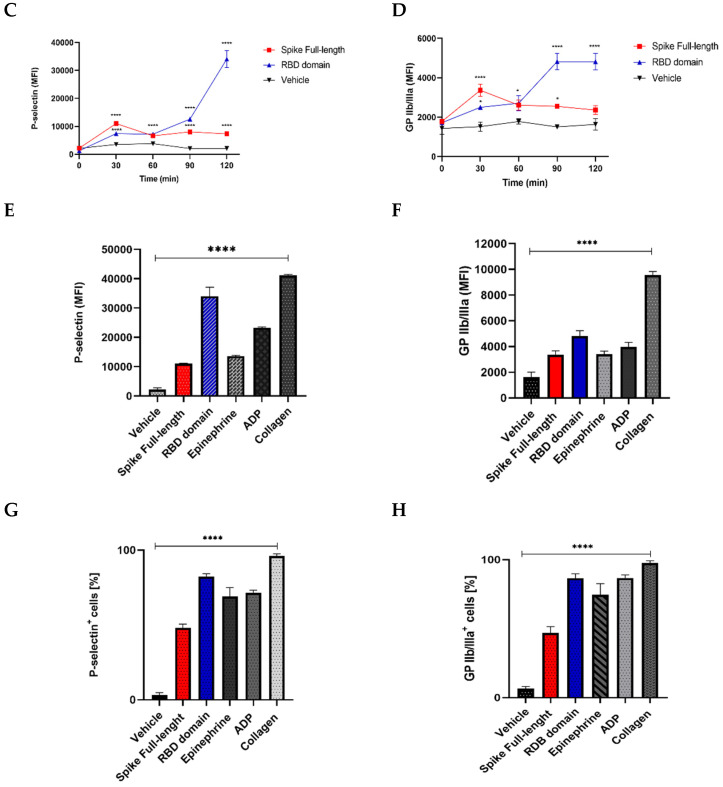
Platelet stimulation with full-length spike protein and RBD domain. Platelet-rich plasma was incubated for 60 min at 37 °C with spike full-length and RBD domain at different concentrations. Mean fluorescence expression of platelet activation markers when incubated with the spike full-length and RBD domain (**A**,**B**). Platelet-rich plasma was incubated for different times at 37 °C with spike full-length and RBD domain at a concentration of 2.0 μg/mL. Tyrode’s buffer was used as control (**C**,**D**). Mean Fluorescence Intensity expression of P-selectin and GP IIbIIIa, respectively, on platelets’ surface when incubated with spike full-length protein, RBD domain, and Tyrode’s buffer at different times (**E**,**F**). Comparison between maximal expression of activation markers P-selectin (CD62) and GP IIbIIIa (PAC1). Spike full-length protein for 30 min (2.0 μg/mL), RBD domain 120 min 2.0 μg/mL, Tyrode’s buffer as vehicle and ADP 20 μM (20 min), epinephrine 100 μM (40 min) and collagen 20 μM (30 min) as known activation agonists (**G**,**H**). Comparison of maximum expression of activation markers indicating the percentages of P-selectin (**G**)- and GP IIbIIIa (H)-positive cells after stimulation with viral proteins spike full-length and RBD domain, known activation agonists and vehicle. *p* values were calculated using one-way ANOVA and Tukey’s post-hoc tests (*n* = 3). Data presented as Mean ± SD. * *p* < 0.05, ****: Statistical difference *p* < 0.0001.

Firstly, we evaluated different concentrations (0.5, 1, and 2 μg/mL) of the full-length (complete) SARS-CoV-2 spike protein and of its RBD domain, and their ability to induce platelet activation. We observed the highest cell activation at a protein concentration of 2 μg/mL. This result coincided for both proteins; therefore, subsequent assays were performed using this same concentration. Mean fluorescence expression of platelet P-selectin and GP IIbIIIa activity are shown in (Figure 1A,B). Then, we explored the full-length spike protein’s and RBD domain’s capacity to activate platelets in a time-dependent manner. The results of fluorescence expression of platelet activation markers P-selectin and GP IIbIIIa are shown in (Figure 1C,D). Compared with the control, the full-length spike protein was able to induce the maximum expression of platelet activation markers at 30 min after stimulation (*p* < 0.0001). Interestingly, the RBD domain induced the higher expression of these proteins at 120 min, and a statistical difference was observed when compared with the unstimulated platelets (*p* < 0.0001). The negative control was treated under the same conditions as the experimental groups but without the stimulation of viral proteins. Finally, we compared the highest expression results of these activation markers for each protein and the percentage of cells positive for the expression of cell activation markers, with the expression of these biomarkers induced by known activation agonists, unstimulated cells, and with the stimuli induced by Tyrode’s buffer used as a vehicle. Our results show that the RBD domain induced a higher expression of both P-selectin and GP IIbIIIa than epinephrine and ADP, but lower than collagen (*p* < 0.0001). Additionally, the expression of activation markers generated by full-length spike protein was similar to that induced by epinephrine but statistically lower than ADP and collagen (*p* < 0.0001). These results are shown in (Figure 1E–H) and confirm that both the spike protein and the RBD domain are able to activate platelets.

### 2.2. Full-Length Spike Promotes Platelet Aggregation in the Presence of Low Doses of Collagen

After we determined that both proteins were able to induce platelet activation, we decided to investigate whether the spike protein or its RBD domain were capable of inducing platelet aggregation, another important function of platelets. 

**Figure 2:**
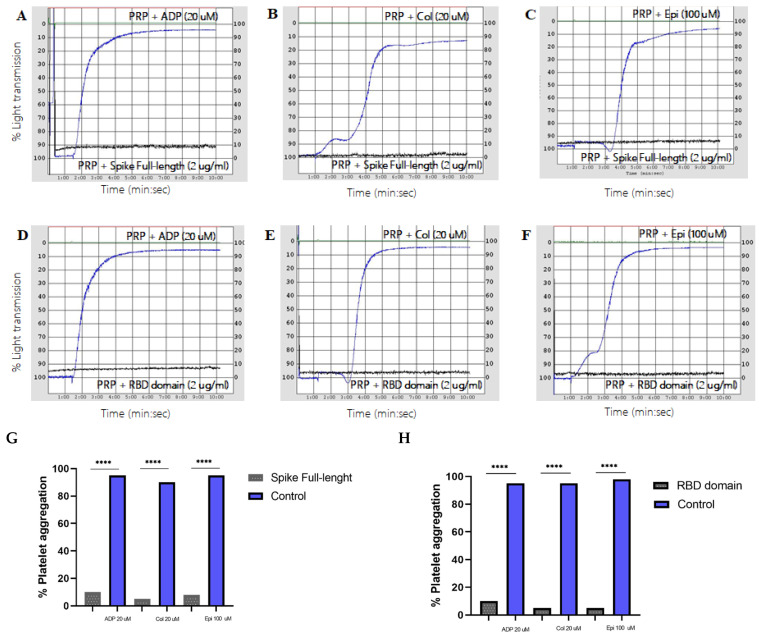
Determination of the aggregation capacity of platelets in response to spike full-length and RBD domain (**A**–**C**). Platelet-rich plasma (PRP) was incubated with spike full-length protein (2.0 μg/mL) for 30 min and compared to positive controls stimulated with a dose of 20 μM ADP, 20 μM collagen, and 100 μM epinephrine (**D**–**F**). PRP was incubated with RBD domain (2.0 μg/mL) for 120 min and compared to positive controls stimulated with a dose of 20 μM ADP, 20 μM collagen, and 100 μM epinephrine (**G**,**H**). Comparison of platelet aggregation induced by known agonists when compared to PRP treated with virus proteins. Platelet agonists, but not proteins, were able to induce greater than 80% of aggregation. *p* values were calculated using one-way ANOVA and Tukey’s post-hoc tests (*n* = 3). Data presented as Mean ± SD; ****: Statistical difference *p* < 0.000.

Our results demonstrate that neither the complete spike protein nor the RBD domain induce platelet aggregation on their own when compared to known aggregation agonists, which achieve aggregation percentages of more than 90% (*p* < 0.0001) (Figure 2A–H). Once we observed that proteins induced activation but not aggregation, based on previous results reported in our lab by García-Larragoiti et al. [22] and in a previous work performed by Chiao-Hsuan et al. [23], we decided to assess if platelet hyperreactivity induced by viral protein stimulation, in addition to subthreshold doses of known aggregation agonists, could restore platelets’ aggregation capacity. 

**Figure 3:**
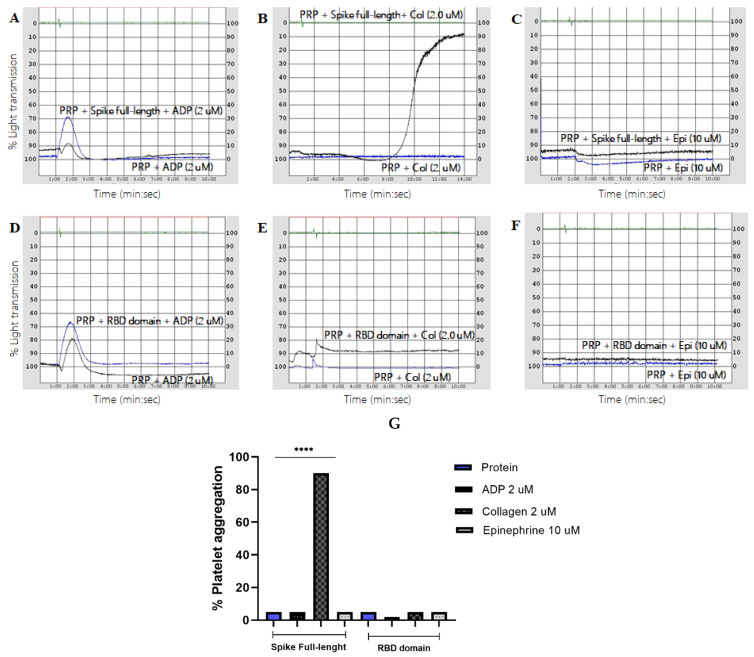
Determination of the aggregation capacity of platelets incubated with spike full-length or RBD domain in response to subthreshold doses of known aggregation agonists (**A**–**C**). PRP was incubated with spike full-length protein (2.0 μg/mL) for 30 min and a dose of 2 μM ADP, 2 μM collagen, and 10 μM epinephrine was added. Only the minimal dose of collagen was able to induce aggregation of PRP when compared to the negative control (PRP + 2 μM ADP, 2 μM collagen, and 10 μM epinephrine, respectively) (**D**–**F**). PRP was incubated with RBD domain (2.0 μg/mL) for 120 min and a dose of 2 μM ADP, 2 μM collagen, and 10 μM epinephrine was added (**G**). Comparison of platelet aggregation induced by minimal doses of known agonists in PRP incubated with full-length spike or RBD proteins when compared to PRP added with minimal doses of known aggregation agonists. None of the known agonists were able to induce aggregation of PRP when compared to the negative control (PRP + 2 μM ADP, 2 μM collagen, and 10 μM epinephrine, respectively). *p* values were calculated using one-way ANOVA and Tukey’s post-hoc tests (*n* = 3). Data presented as Mean ± SD. ****: Statistical difference *p* < 0.0001.

Interestingly, our results shown in Figure 3 prove that minimal doses of collagen were able to re-establish platelet aggregation up to 90% in cells previously treated with the full-length spike protein (*p* < 0.0001) (Figure 3B); however, this was not observed in assays to which ADP or EPI were added (Figure 3A–C). On the other hand, as is shown in (Figure 3D–F), none of the minimal doses of known agonists were able to induce platelet aggregates in those cells previously stimulated with the RBD domain. Our results suggest that SARS-CoV-2 full-length proteins induce a hyperreactive state in platelets forming aggregates when exposed to aggregating factors. 

### 2.3. The Spike Protein and Its RBD Domain Stimulates Platelets’ Release of Pro-Inflammatory Factors

**Figure 4:**
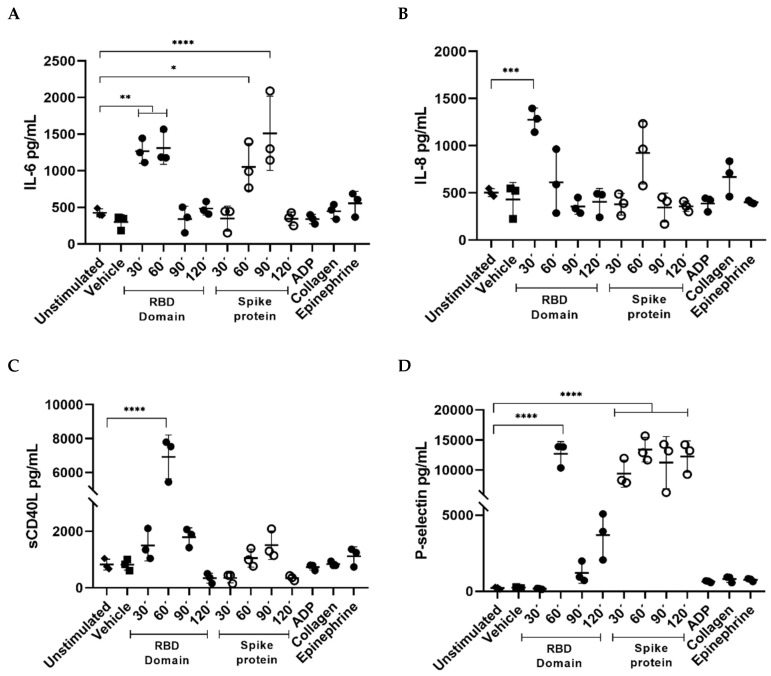

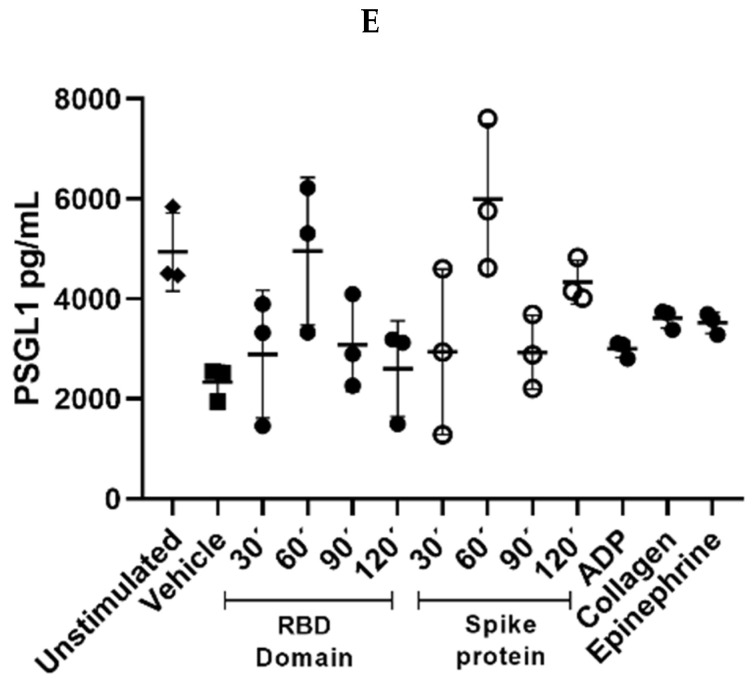
The spike protein and its RBD domain stimulate platelets’ release of pro-inflammatory factors. Comparison of supernatant concentration of (**A**) IL-6, (**B**) IL-8, (**C**) sCD40L, (**D**) P-selectin and (**E**) PSGL-1 in PRP incubated with spike full-length protein (2.0 μg/mL) and RBD domain (2.0 μg/mL) at different times. Known platelet aggregation agonists were used as activation controls (20 μM ADP, 20 μM collagen, and 100 μM epinephrine), Tyrode’s buffer was used as vehicle and PRP treated under the same conditions but without external stimuli as a negative control. *p* values were calculated using one-way ANOVA and Tukey’s post-hoc tests (*n* = 3). Data presented as Mean ± SD. Statistical difference *p < 0.05, ** *p* < 0.01, *** *p* < 0.001, ****p < 0.0001.

In order to approach the role of platelets in response to viral proteins, we decided to assess the concentration of different biomarkers related to inflammation in the supernatant of PRP stimulated with each SARS-CoV-2 protein at different times. We found that IL-6 concentration was higher in PRP after stimulation for 30 and 60 min (min) with the RBD domain when compared with unstimulated PRP (*p* < 0.01). Interestingly, full-length spike protein shows a raise in IL-6 concentration at 60 min (*p* < 0.05), presenting the main pick at 90 min (*p* < 0.0001) when compared with controls (Figure 4A). Moreover, only RBD was able to induce platelet release of IL-8 after a stimulus of 30 min (*p* < 0.001). sCD40L at 60 min of stimulation with the RBD domain showed differences in concentration when compared to control (*p* < 0.0001) (Figure 4B,C). Finally, we observed that both proteins stimulated the significant release of P-selectin, but not the expression of PSGL-1 (Figure 4D,E). Significant RBD-induced P-selectin release was observed at 60 min (*p* < 0.0001), while the full-length spike protein maintained significant concentrations during 30, 60, 90, and 120 min, when compared with unstimulated PRP (*p* < 0.0001). Curiously, none of the known activation agonists such as ADP, EPI or collagen showed an increase in the concentration of inflammatory-related biomarkers. These results suggest that platelets may contribute to the cytokine storm reported in SARS-CoV-2 infection by showing a pro-inflammatory phenotype. 

### 2.4. Platelets Contribute to a Procoagulant Microenvironment when Stimulated with Full Length Spike Protein and RBD Domain

**Figure 5:**
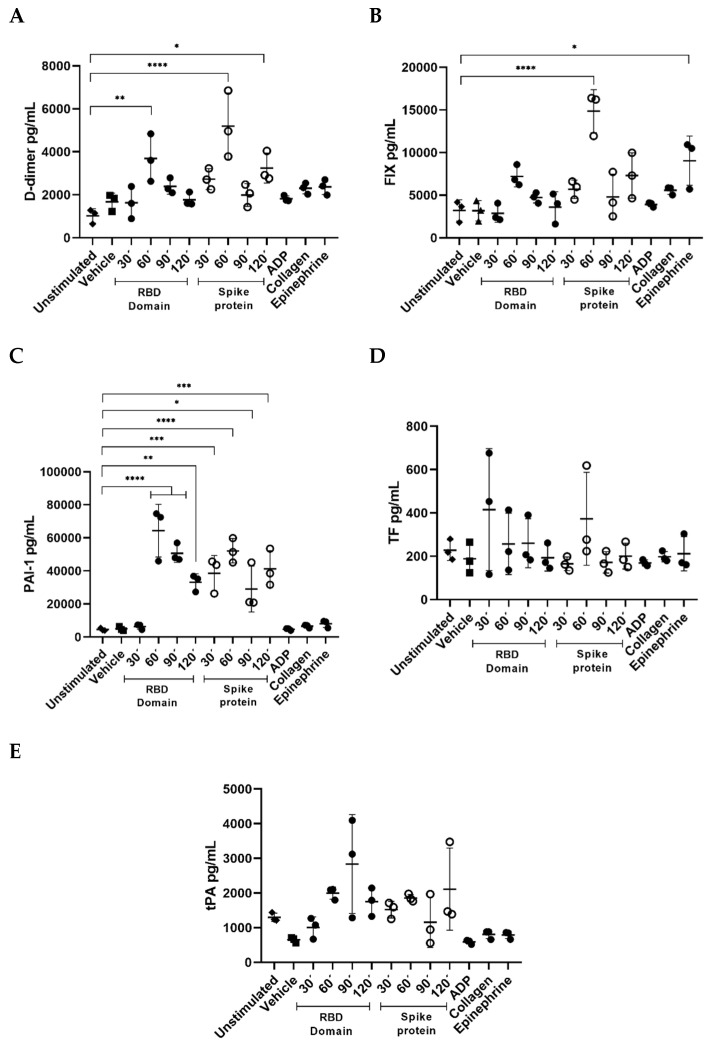
The spike protein and its RBD domain stimulate platelets’ release of pro-inflammatory factors. Comparison of supernatant concentration of (**A**) D-dimer, (**B**) FIX, (**C**) PAI-1, (**D**) TF and (**E**) tPA in PRP incubated with spike full-length protein (2.0 μg/mL) and RBD domain (2.0 μg/mL) at different times. Known platelet aggregation agonists were used as activation controls (20 μM ADP, 20 μM collagen, and 100 μM epinephrine), Tyrode’s buffer was used as vehicle and PRP treated under the same conditions but without external stimuli as a negative control. *p* values were calculated using one-way ANOVA and Tukey’s post-hoc tests (*n* = 3). Data presented as Mean ± SD. Statistical difference * *p <* 0.05, ** *p* < 0.01, *** *p* < 0.001, **** *p* < 0.0001.

Hemostasis and fibrinolysis processes are altered in severe COVID-19, and platelets are an important source of coagulation and fibrinolytic factors. In order to measure whether protein-stimulated platelets contribute to this misbalance, we determined five different biomarkers related to a prothrombotic state. D-dimer concentrations were found to be significantly higher in platelets stimulated with the RBD domain for 60 min (*p* < 0.01) and with the full-length protein at 60 min (*p* < 0.0001) (Figure 5A). Moreover, only the full spike protein induced a significant increase in Factor IX concentrations after 60 min of stimuli (*p* < 0.0001). Only EPI showed increments of FIX (*p* < 0.05) (Figure 5B). Interestingly, both full-length spike protein and RBD domain induced a time-dependent release of PAI-1, 60 min being the time where the highest concentrations were found (*p* < 0.0001). (Figure 5C). We did not find differences in TF and tPA at any time (Figure 5D,E). These results indicate that platelets release coagulation factors that contribute to a prothrombotic state when exposed to an immune insult, such as that reported in COVID-19. 

## 3. Discussion

Platelets can be found in large numbers in peripheral blood, and due to their functional characteristics, these cells can play an important role in the immune response. Platelet count in COVID-19 varies according to disease severity. Severe thrombocytopenia is rarely reported in COVID-19 patients and correlates with greater morbidity/mortality [24]. Moreover, mild thrombocytopenia has been detected in most cases. This drop in platelet count may be related to a worsening thrombotic state [25,26]. However, information on the ability of structural proteins and their domains to activate platelets is not entirely clear. Regarding the interaction of these viral proteins with platelets in this study, we demonstrated using flow cytometry that full-length spike protein and RBD can induce platelet activation and degranulation. Initially, we observed that the full-length spike protein and the RBD domain induced the highest platelet activation at a concentration of 2 μg/mL. Our results concur with those reported by Zhang et al., who also used this concentration to perform platelet stimulation assays with spike protein and the S1 subunit of the protein [20]. Both the full protein and its domain were able to induce high expression of P-selectin and the glycoprotein IIbIIIa, known markers of platelet degranulation and activation, respectively [10,27]. Grobbelaar et al. previously reported that the spike subunit 1 (S1) of SARS-CoV-2 can induce fibrin resistance to fibrinolysis and induce platelet activation, contributing to clot formation [28]. We are one of the first groups to report the ability of the RBD domain to induce platelet activation.

However, there is no consensus on the hypothesis that platelet activation in SARS-CoV-2 is due to the ACE2 receptor [20,21,29,30,31]. It has been reported that platelets do express ACE2 and that spike protein binding induces platelet activation and degranulation with the increased surface expression of P-selectin. Moreover, the expression of GP IIbIIIa induced by SARS-CoV-2 virions, contributing to enhanced thrombosis in COVID-19, has also been reported [20]. On the other hand, other groups have proposed different mechanisms of platelet activation in COVID-19 based on the expression of platelet EMMPRIM (CD147) [21], ACE2-independent mechanisms [29,30], and the presence of aberrant glycosylation in antibodies against spike [31]. Our results confirm that the spike full-length protein and the RBD actively participate in SARS-CoV-2 immunopathogenesis by inducing platelet activation and degranulation. This process may be mediated by not only one receptor but by the participation of different platelet surface membrane proteins. Further molecular docking studies may be useful to evaluate the variety of possible receptors on the platelet membrane surface to the spike protein and domain.

Platelet physiology indicates that activation is followed by an aggregation process that contributes to microthrombi formation. In order to address whether proteins can induce platelet aggregates, we incubated the platelets with the SARS-CoV-2 proteins. Our aggregation results indicate that these proteins cannot induce platelets’ aggregation, but we report that platelets that were incubated with full-length spike protein and stimulated with low doses of collagen recovered aggregation capacity. This concurs with the results of Zhang et al., which showed that cells prestimulated with the spike protein or its subunits induce a hyperreactive state in platelets that can aggregate when low doses of known agonists are added [20]. Furthermore, there is evidence that platelets’ responses to known aggregation agonists differ depending on the agonist and the dose used [32,33]. Differences in inducing platelet phenotype by virions, proteins, or domains may be due to alterations in cell proteome, which may explain why we observed recovery of aggregation when a strong agonist like collagen is used. This behaviour has been previously reported with other viral proteins. Garcia-Larragoiti et al. found that dengue virus non-structural protein 1 (NS1) induces platelet reactivity that favors the formation of platelet aggregates when low doses of epinephrine are added [22]. 

Hyperactivation of platelets in SARS-CoV-2 infection may not only contribute to thrombotic risk, but also to the inflammation process. COVID-19 patients with worse outcomes present high plasmatic levels of inflammatory cytokines and pro-thrombotic factors [33,34]. Platelets represent a rich source of these factors, since they contain granules with large amounts of molecules participating in inflammatory and hemostasis processes. Our results demonstrate that full-length spike protein and its RBD domain promote the release of pro-inflammatory factors in a time-dependent manner. We found differences in platelet response when comparing an inflammatory insult with an aggregating stimulus. This may be regulated by signaling pathways activated by platelets according to the stimulus that induces their activation [35]. It is known that the kinetics and concentration of molecules released by platelets depend on the activation agonist used, as well as the concentration of the same. Differences in the amount of these biomolecules released may be related to a selective degranulation process where these are stored, and our results contribute to the hypothesis of the presence of distinct subpopulations of α-granules in platelets [36,37]. On the other hand, our results are similar to those found by van Holten et al., who observed using immunoassays and proteomic assays that there is no homogeneous release profile of platelet granular content. Rather, it is a heterogeneous process that depends on the characteristics of the activation agonist used [38]. Variations in the concentrations of thrombotic inflammatory factors released by platelets following stimulation with known viral proteins and aggregation agonists may be associated with different aspects. The half-life of some of these factors is only a few minutes. Subsequent denaturation of these factors may be mediated by enzymatic mechanisms. Another important factor is the possible presence of soluble cytokine receptors, generated by proteolytic cleavage of membrane receptors upon activation of the cells that express them. These receptors act as competitive inhibitors of these factors, decreasing their concentration [39]. In this area, we report that SARS-CoV-2 proteins induce the release of anti-fibrinolytic and pro-thrombotic factors by platelets. These results confirm that viral proteins in COVID-19 are able to induce a pro-inflammatory and pro-coagulant phenotype in platelets that may contribute to an immunothrombotic process. Our result concurs with previously reported works where platelets showed a procoagulant phenotype in COVID-19 [20,36,40]. On the other hand, platelets’ differential response to other viruses has been reported. Assinger et al. demonstrated that human cytomegalovirus–platelet interaction induced a pro-inflammatory and proangiogenic response [41]. 

In conclusion, in this work we demonstrate the ability of platelets to actively participate in the immune response against SARS-CoV-2 structural proteins. These results sustain platelet’s role in the development of a pro-inflammatory and pro-coagulant state. We found that platelets have a granular content release response that is differential according to the stimuli received. The response by platelets directed against the viral stimulus also favors the activity of other platelets, perpetuating this response. Our results confirm that platelets are important cells in the response against pathogenic agents; however, it is important to carry out more studies on the physiological response of platelets to pathological agents in the face of the immunological challenges that constantly arise today.

## 4. Materials and Methods

### 4.1. SARS-CoV-2 Spike Full-Length and RBD Domain Proteins

Recombinant SARS-CoV-2 full-length protein was provided by Bio Vision Human CellExp^®^ (Waltham, MA, USA). Cat # P1525, Size: P1525-50 SARS-CoV-2 Spike protein, and the SARS-CoV-2 Spike protein RBD was provided by GenScript^®^ (Piscataway, NY, USA). Cat # Z03491 SARS-CoV-2 spike protein (RBD, mFc Tag).

### 4.2. Blood Sample Collection

Whole human blood was obtained from healthy volunteers by clean venipuncture after signing the informed consent form. Healthy donors were individuals within a normal range of body mass index that were not taking any kind of medication and did not exhibit any abnormal findings on routine blood chemistry and hematological tests. All experiments were performed on donors who did not present previous exposure to SARS-CoV-2, previous exposure of at least 1 year, or exposure to SARS-CoV-2 vaccines, to ensure the absence of antibodies to the virus or its viral proteins that could interfere with platelet stimulation assays. Blood was obtained by clean venipuncture and the first tube (2 mL) of blood was discarded to ensure the accuracy of platelet testing. Samples were collected in vacutainer tubes with 3.2% (0.109 mol/L) sodium citrate solution as an anticoagulant (Becton Dickinson) and processed within 4 h after collection.

### 4.3. Platelet Rich Plasma Isolation

Platelet-rich plasma (PRP) was obtained by centrifuging at 100× *g* for 10 min at room temperature (RT). The supernatant was carefully collected to avoid disrupting the buffy coat and it was left to rest for 30 min in darkness before analysis by flow cytometry. 

### 4.4. Platelet Stimulation with Spike SARS-CoV-2 and RBD Domain

Platelet-rich plasma was adjusted to a concentration of 1 × 10^7^ with Tyrode’s buffer to maintain the integrity of the platelets. They were incubated directly with SARS-CoV-2 full-length spike protein and RBD for different concentrations (0.5, 1.0, and 2.0 μg/mL) to determine the best protein concentration to stimulate platelets. Subsequently, PRP was stimulated with viral proteins at a concentration of 2 µg/mL for a minimum of 30 min to a maximum of 120 min at a temperature of 37 °C in individual assays. Platelet activation was confirmed by activation of the heterodimeric complex GP IIbIIIa (PAC1-FITC) and expression of P-selectin (CD62-PE) determined by flow cytometry. The supernatant was obtained after stimulation and stored at −70 °C until use for immunothrombotic biomarker determination. Assays were designed based on previous published studies using different concentrations of SARS-CoV-2 spike full-length protein, its domains and subunits, as well as different incubation times [20,21,28,42].

### 4.5. Flow Cytometry Assays

CD41/PECy7 (BioLegend Cat. No. 303718) was used as an identity marker for platelets, PAC-1/FITC (BioLegend Cat. No. 362804) for glycoprotein GP IIbIIIa and CD62/PE (BioLegend Cat. No. 304906) for P-selectin were used as activation markers. IgG1 k (BioLegend Cat. No. 400125), FITC Mouse IgM k Isotype (BioLegend Cat. No. 401605) and Mouse IgG1 k Isotype (BioLegend Cat. No. 400111) were used as isotype control, respectively. The gating strategy of the cell populations was performed according to previously reported by the research group in García-Larragoiti et al. [22]. Dark conditions and minimal handling were used during the assay to avoid external activation of platelets. Adenosine Di Phosphate (ADP) (20 µM) for 20 min, collagen (20 µM) for 30 min, and epinephrine (EPI) (100 µM) for 40 min were used as positive platelet activation controls [27]. Concentrations were used following the instructions suggested by the supplier PAR/PAK II^®^ BIO/DATA CORPORATION (Horsham, PA, USA). The acquisition was performed in a CytoFLEX, BECKMAN COULTER^®^ (Brea, CA, USA). Results were analyzed using FlowJo v 10.8.0. 

### 4.6. Determination of Immunothrombotic Biomarkers in Platelet Stimulated Supernatant

As previously described, PRP stimulated within viral proteins during different times was carefully collected with a pipette. Separate assays of PPR treated with ADP (20 µM) for 20 min, collagen (20 µM) for 30 min, epinephrine (100 µM) for 40 min, Tyrode’s buffer, and unstimulated PRP were used as controls. A panel of 10 thrombotic-inflammatory biomarkers was analyzed: Interleukin 6 (IL-6), Interleukin 8 (IL-8), P-selectin, P-selectin ligand 1 (PSGL-1), sCD40L, D-dimer, tissue plasminogen activator (tPA), tissue plasminogen activator inhibitor 1 (PAI-1), tissue factor and coagulation factor IX. Determination of these biomarkers was performed by flow cytometry using the BioLegend^®^ (San Diego, CA, USA) LEGENDplex Kit TM Human Thrombosis Panel Standard following the instructions suggested by the supplier. Samples were read in a CytoFLEX, BECKMAN COULTER^®^. Briefly, samples were incubated with beads that differed in size for 2 h. Each bead group was conjugated with specific antibodies on its surface that captured a specific analyte. After washing, a cocktail of biotinylated detection antibodies was added for 1 h, forming a detection complex between the capture bead–analyte–detection antibody. Finally, Streptavidin-phycoerythrin (SA-PE) was added and incubated for 30 min. Samples were taken to the CytoFLEX cytometer for analysis. 

### 4.7. Light Transmission Aggregation Assays

Whole blood was obtained from healthy donors who did not present previous exposure to SARS-CoV-2 or previous exposure of at least 1 year in tubes with sodium citrate (3.2%). PRP was obtained under the conditions described above and incubated for 30 min with SARS-CoV-2 full-length spike protein and for 120 min with RBD both at a concentration of 2 µg/mL in separate assays. Platelet-poor plasma (PPP) was separated by centrifugation at 2500× *g* for 15 min and used as a blank. Subsequently, 0.5 mL of the PRP previously incubated with the SARS-CoV-2 proteins and 0.5 mL of unstimulated PRP were placed in the cuvette of the aggregometer, containing a siliconized magnetic bar, at a constant temperature of 37 °C; the results were compared against positive controls (PRP + ADP 20 µM, PRP + Collagen 20 µM, PRP + Epinephrine 100 µM). To investigate if the addition of minimal doses of known aggregation agonists in platelets previously treated with SARS-CoV-2 proteins could improve platelet aggregation, doses of ADP (2.0 µM), collagen (2.0 µM), and epinephrine (10 µM) were added. Assays were performed by triplicate. Light transmission was measured on a Chronolog 560ca aggregometer (Chrono-log). All data were analyzed with AGGRO/LINK^®^8 software.

### 4.8. Statement of Ethics

All the studies with healthy donors were approved by the ethics committee of the Faculty of Medical and Biological Sciences “Dr. Ignacio Chávez” of the UMSNH registration number 004/P/5/2021 and the General Hospital “Dr. Miguel Silva”; registration number CEI/2021/III-269. Morelia, México.

### 4.9. Statistical Analysis

Comparisons between two groups were made with an unpaired *t*-test. Multiple group comparisons were made using a one-way analysis of variance. Tukey’s test was used as a post hoc test for pairwise comparisons. The data that were not normally distributed were analyzed using nonparametric statistical analysis. All experiments were independently repeated three times. *p* < 0.05 was considered statistically significant. All data were presented as means ± standard deviation. Statistical analysis was performed using GraphPad Prism 7 (GraphPad Software, Inc, San Diego, CA, USA).

## Data Availability

Not applicable.
